# Physical, Chemical, Microbiological and Sensory Characteristics of a Probiotic Beverage Produced from Different Mixtures of Cow's Milk and Soy Beverage by *Lactobacillus acidophilus* La5 and Yoghurt Culture

**DOI:** 10.17113/ftb.57.04.19.6344

**Published:** 2019-12

**Authors:** Edina Šertović, Zlatan Sarić, Miroljub Barać, Irena Barukčić, Aleksandar Kostić, Rajka Božanić

**Affiliations:** 1 University of Bihać, Biotechnical Faculty, Luke Marjanovića bb, 77000 Bihać, Bosnia and Herzegovina; 2 University of Sarajevo, Faculty of Agriculture and Food Sciences, Zmaja od Bosne 8, 71000 Sarajevo, Bosnia and Herzegovina; 3 University of Belgrade, Faculty of Agriculture, Nemanjina 6, 11080 Beograd, Serbia; 4 University of Zagreb, Faculty of Food Technology and Biotechnology, Pierottijeva 6, 10000 Zagreb, Croatia

**Keywords:** cow's milk, soy beverage, probiotics, *Lactobacillus acidophilus*, fermentation

## Abstract

The aim of this paper is to determine nutritive, functional, microbiological and sensory properties of probiotic beverages produced from different volume ratios of cow's milk and soy beverage (25:75, 50:50 and 75:25). Pure cow’s milk and soy beverage served as control samples. Fermentation was performed at 43 °C by a combined culture consisting of the probiotic strain *Lactobacillus acidophilus* La5 and yoghurt culture. Viable counts of La5 strain in the produced beverages ranged from 7.52 to 8.20 log CFU/mL, which is above the probiotic minimum (10^6^ CFU/mL). Lactic acid was the most prevalent organic acid in all samples (660.1 to 1003.0 mg/100 mL). The fatty acid profiles of fermented beverages were as follows: the mass fraction of saturated fatty acids was 22.2-82.7%, of unsaturated fatty acids 22.3-77.8% and of polyunsaturated fatty acids 15.5-65.9%. The main soy sugars were transformed well (80% stachyose and 50% raffinose conversion) into lactic acid during fermentation. Functional probiotic beverages were successfully produced from different volume ratios of cow's milk and soy beverage by *L. acidophilus* La5 and yoghurt culture. Mixing cow's milk with soy beverage significantly improved the sensory properties of the product, especially its smell, taste and colour. The acceptability test showed good acceptance by potential consumers of all fermented beverage samples except for the sample made from 100% soy beverage. In the end, the obtained results represent a good basis for further optimisation of the ideal volume ratios of cow's milk and soy beverage for production of fermented beverages characterised by good viability of probiotic bacteria as well as by good functional, nutritive and sensory characteristics.

## INTRODUCTION

Soy beverage is a traditional oriental drink. It is an aqueous extract of soybean grain, which can be a good substitute for cow's milk. Among the most limiting factors of the use of soy beverage is the presence of considerable amounts of non-digestible oligosaccharides as well as an unpleasant odour and the taste that can be caused by lipoxygenase, the enzyme from soybean grain. Probiotics are commonly defined as mono- or mixed cultures of living microorganisms, which, when used by humans or animals, have beneficial effects on the host, improving the properties of the existing microflora. The most commonly used probiotic strains belong to genera *Lactobacillus* and *Bifidobacterium* (*1*). *Lactobacillus acidophilus* and *Lactobacillus casei* are considered as the most important probiotic species, and are believed to have positive effects on human health (*2*). The survival and the viable cell counts of probiotic strains in the final product at the moment of consumption are their most important qualitative parameters. Although there is no universal agreement regarding the recommended level, the values of 10^6_^10^8^ CFU/mL are generally accepted as minimum and satisfactory levels (*3*). The fermentation of soy beverage with probiotic bacteria improves the nutritional value of these products and allows food to function as a supply of probiotic organisms to consumers. Several studies have focused on investigating the growth of probiotic bacteria in soy beverage compared to cow's milk. However, there is a small number of studies related to fermented products with probiotics in combination with yoghurt bacteria and their behaviour in various combinations of soy beverage and cow's milk. Fermentation of soy beverage offers the possibility to transform and improve the taste and texture (*4*, *5*). Therefore, this research aims to determine the effect of combining cow’s milk and soy beverage with probiotic bacteria (*L. acidophilus*) and to evaluate the physicochemical, microbiological and sensory characteristics of fermented probiotic soy-based beverages, as well as their acceptability at the end of fermentation.

## MATERIALS AND METHODS

### Raw materials and dairy starter cultures used for beverage production

Homogenized, ultra-high-temperature (UTH) sterilised cow’s milk with 2.50% fat (Meggle, Bihać, Bosnia and Herzegovina) and soy beverage with 1.90% fat (dmBio; dm-drogerie markt GmbH & Co. KG, Karlsruhe, Germany) were used to produce probiotic drinks. Physical, chemical and microbiological characteristics of milk samples were in accordance with the standards. Yoghurt culture YF-L811 (Christian Hansen, Hørsholm, Denmark) and the probiotic strain *L. acidophilus* La5 (Christian Hansen) were used for the fermentation of different mixtures of cow's milk and soy beverage.

### Production of fermented beverages

Five different volume ratios of homogenized milk and soy beverage (100:0, 75:25, 50:50, 25:75 and 0:100) were prepared by mixing UHT-sterilised milk and soy beverage. Samples were inoculated with probiotic starter culture (*L. acidophilus* La5) and yoghurt culture YF-L811. The inoculum was first made by mixing 0.1 g probiotic culture (strain La5; Christian Hansen) and 0.07 g yoghurt culture (YF-L811; Christian Hansen) in 100 mL of milk. For each of the five different samples, a special inoculum was prepared. Every inoculum was incubated for 30 min at 43 °C to adapt the bacteria to the medium. After incubation, the inoculum was cleaved into milk samples intended for the production of probiotic beverages. Fermentation was carried out at 43 °C until pH reached the value of 4.6. Characteristics of the obtained probiotic beverages were monitored during and at the end of the fermentation. Three repeated batch fermentations were performed.

### Microbiological analysis

The viable cell counts of the probiotic strain in the produced samples were determined by a standard pour plate method using de Man, Rogosa and Sharpe (MRS) agar (Merck, Darmstadt, Germany) supplemented with clindamycin (Sigma-Aldrich, Merck, Taufkirchen, Germany). Following ISO 20128:2006(E)/IDF 192:2006(E) standard (*6*), clindamycin was added to a sterilized MRS agar cooled to 43 °C just before pouring it into the Petri dish to prevent the growth of the used yoghurt culture during fermentation. Subsequently, Petri dishes were incubated at 37 °C for 72 h, after which colonies of probiotic bacteria (strain La5) were enumerated. The obtained data represent the arithmetic average of the enumerated colonies expressed as CFU/mL.

### Physical, chemical and sensory analyses

The chemical composition and acidity of the produced probiotic beverages at the end of fermentation were determined by standard analytical methods. On the first day after the production of probiotic beverages, viscosity, sensory properties and product acceptability were monitored. Rotary rheometer Rheomat RM180 (Rheometric Scientific, Inc., Piscataway, NJ, USA) was used to determine the rheological properties of probiotic beverage samples at 20 °C and a shear rate of 100 to 1290 s^-1^. Sensory properties were rated by a weighted scoring method ISO 22935-3:2009/IDF 99-3:2009 (*7*) by a group of five trained female sensory analysts (30–50 years old). Acceptability of probiotic beverages was evaluated by testing 30 young consumers (students, male and female around 20 years old) using the verbal 9-point hedonic scale (Peryam) (*8*).

### Determination of sugar in probiotic beverages

The sugar from the samples of fermented cow's milk and soy beverage was extracted using a method previously described by Scalabrini *et al*. (*9*) with some modifications. An aliquot of 3 mL of the sample was taken and centrifuged (centrifuge series SL16; Thermo Scientific, San Jose, CA, USA) at 14 000×*g* for 30 min to remove proteins. Then, the content was filtered. Plastic injection and membrane filters with ≤0.20 μm pore size were used for filtration. Sugar content was analyzed using the high-performance liquid chromatography (Agilent 1260; Agilent Technologies, Waldbronn, Germany) equipped with Alltima amino column (250 mm×4.6 mm×5 μm; Hichrom Limited, Lutterworth, UK). The obtained filtrate of 20 μL was injected directly using the autosampler at a flow rate of 1 mL/min at room temperature for 15 min. The volume ratio of acetonitrile and water 75:25 was used as a mobile phase with isocratic flow.

### Determination of organic acids

Organic acids in fermented probiotic products after fermentation were determined by high-performance liquid chromatography (HPLC) using the method of Shah and Ravula (*10*). A volume of 3 mL of yoghurt was mixed with 50 μL of 15.5 M nitric acid and 1 mL of 0.01 M sulfuric acid. The sample was centrifuged (centrifuge series SL16; Thermo Scientific) at 14 000×*g* for 30 min to remove the proteins and the content of individual organic acids was then analyzed by HPLC (Agilent 1260; Agilent Technologies) equipped with Alltima amino column (250 mm×4.6 mm×5 μm; Hichrom Limited). The experiment was repeated three times. Organic acids were identified by comparing their retention times with standard solutions of lactic, citric and acetic acids.

### Fatty acid determination using gas chromatography

The lipid compounds were dissolved in 1 mL of hexane and converted into fatty acid methyl esters (FAME) according to Barać *et al.* (*11*). Gas chromatographic analysis of FAME was carried out on an Agilent 6890A (Agilent Technologies, Santa Clara, CA, USA) with flame ionization detector (FID) and column Supelco SP-2560 (100 m×0.25 mm, stationary phase thickness 0.20 μm; Merck, Bellefonte, PA, USA). Peaks of individual FAME were identified by comparing its retention time with the retention time of the mixture of 37 standards (Supelco 37 component FAME mix, Merck, Bellefonte, PA, USA). Each analysis was performed in triplicate, and the fatty acid content was calculated in mg/g of lipids and expressed in relative amounts as mass fraction of total fatty acids.

### Lipid quality indices

The ratio of unsaturated/saturated fatty acids (UFA/SFA) as well as desirable fatty acids (DFA) was calculated from the fatty acid profile of the probiotic beverages of cow's milk and soy beverage. DFA were calculated according to the following equation:

DFA=ΣMUFA+ΣPUFA+C18:0 /1/

where MUFA and PUFA are mono- and polyunsaturated fatty acids and C18:0 is stearic acid. Furthermore, in order to link the fatty acid profiles with the risk of cardiovascular disorders, the atherogenic index (AI) and thrombogenic index (TI) (*11*) were calculated according to the following equations:

AI=[(4·C14:0)+C16:0+C18:0]/ΣMUFA+ΣPUFA /2/

and

TI=(C14:0+C16:0+C18:0)/(0.5 MUFA+0.5 PUFA-n6+3 PUFA-n3+PUFA-n3/PUFA-n6) /3/

where C14:0 is tetradecanoic acid and C16:0 is palmitic acid. AI denotes the relationship between the sum of the main saturated fatty acids and the main classes of unsaturated fatty acids. The former is considered as proatherogenic (giving priority to lipid adhesion to the circulatory and immune system cells), and the latter antiatherogenic (inhibiting plaque aggregation and reducing cholesterol levels, esterified fatty acids and phospholipids, thereby preventing the appearance of micro- and macrovascular diseases) (*11*). TI expresses the tendency of creating clots in the blood vessels. It is determined as the ratio between prothrombogenetic (saturated) and antithrombogenic fatty acids (MUFAs, PUFAs-n6 and PUFAs-n3).

### Sodium dodecyl sulfate-polyacrylamide gel electrophoresis

The polypeptide composition of protein extracts was determined by sodium dodecyl sulfate-polyacrylamide gel electrophoresis (SDS-PAGE) according to Fling and Gregerson (*12*). Molecular mass of polypeptides was determined using low-molecular-mass standards (Pharmacia, Uppsala, Sweden). The molecular mass of the markers included phosphorylase B (94.0 kDa), bovine serum albumin (67.0 kDa), ovalbumin (43.0 kDa), carbonate anhydrase (30.0 kDa), soybean trypsin inhibitor (20.1 kDa) and α-lactalbumin (14.4 kDa). The concentration of polypeptides was quantified densitometrically. The gels were scanned with the Mustek 12000 SP (Mustek Europe B.V., Neuss, Germany) PC scanner and were quantified by SigmaGel software v. 1.1 (*13*) that enabled automatic integration. The content of individual polypeptides is expressed in relation to the total surface area of the peaks detected in the sample, namely, as the percentage of the polypeptide content, from the content of the total protein extraction in one sample obtained by gel electrophoresis. All samples were analyzed in triplicate.

### Statistical analysis of the results

The results of the analyzed samples are shown as the mean value±standard deviation. One-way analysis of variance (ANOVA) and multiple comparisons (Duncan's *post-hoc* test) were used to estimate significant difference in data at the significance level of p<0.05. Statistics was implemented using Microsoft Office 2014 and demo versions of the MS Office XLSTAT-Pro 2014 (*14*) statistical package. The principal component analysis (PCA) analysis was also done.

## RESULTS AND DISCUSSION

### Monitoring the flow of fermentation of probiotic beverages

**Fig. 1:**
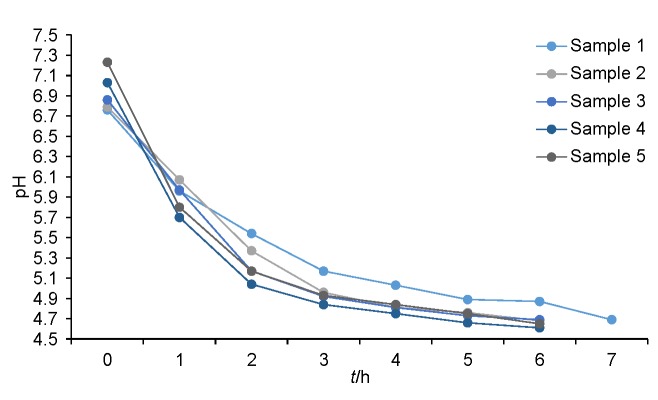
Changes in pH value during milk fermentation by *Lactobacillus acidophilus* (La5) with yoghurt culture at 43 °C. Samples (in %): 1=cow's milk 100, 2=cow's milk 75+soy beverage 25, 3=cow's milk 50+soy beverage 50, 4=cow's milk 25+soy beverage 75, 5=soy beverage 100. The data are mean value of *N*=3

The fermentation of milk and soy beverage samples (100:0, 0:100, 25:75, 50:50, 75:25) with probiotic strain La5 and starter culture lasted 5 to 7 h. Fig. 1 shows changes in the pH value during fermentation in all analyzed samples.

A decrease in the pH value of sample 1 (100% cow's milk) at the beginning of fermentation was rather slow, probably due to the presence of cow’s milk proteins with higher buffer capacity than that of soy protein (*15*, *16*). In general, it could be noticed that increasing soy beverage volume ratio was accompanied by a decrease in pH and increase of acidity, which could be attributed to the significantly reduced buffer capacity (*17*, *18*). This justifies the fact that the fermentation of sample 1 (100% cow's milk) lasted longer than of other samples with equal fermentation time. The addition of soy beverage to cow's milk resulted in an increase in the rate of pH drop during fermentation and increase in acidity.

### Microbiological characteristics of the product at the end of the fermentation

In order to consider a product as a probiotic, it must contain a minimum of 10^6^ live probiotic cells per mL (CFU/mL) (*1*). The growth of probiotic bacteria in cow’s milk combined with soy beverage can be influenced by the presence of free amino acids, other bacteria, the formation of hydrogen peroxide, and the concentrations of lactic and acetic acids (*19*, *20*). For growth and reproduction, lactobacilli require certain conditions such as low oxygen concentration, and fermentable carbohydrates, proteins, vitamin B, unsaturated fatty acids and minerals. At the end of fermentation, the average number of lactobacilli in fermented beverages from cow’s milk combined with soy beverage shows that there is no statistically significant difference among samples in the number of probiotic bacteria at the end of fermentation (data not shown). The number of probiotic bacteria *L. acidophilus* La5 in the samples of produced beverages ranged from 7.52 to 8.20 log CFU/mL.

Although a significant pH decrease occurs, it did not significantly affect the survival of lactobacilli. A sudden increase in yogurt acidity reduces the ability of probiotic bacteria to survive (*21*). Daneshi *et al*. (*22*) found that the storage temperature was important for the survival of lactobacilli in yoghurt, and it was shown that 2 °C is the ideal temperature for storing this product. Considering the obtained results (7.52 to 8.20 log CFU/mL), it can be said that probiotic bacteria (strain La5) grow equally well in all samples, regardless of the type of milk or their ratio. The presence of oligosaccharides that can act as prebiotics in soy beverage contributes to *Lactobacillus* growth, but the soy beverage contains amino acids and peptides that also stimulate the growth of probiotic bacteria (*5*).

### Physical and chemical characteristics

Table 1 shows physical and chemical characteristics of the samples of fermented probiotic beverages at the end of fermentation. The results show that among the dairy products obtained by fermentation with strain La5, sample 1 (100% cow's milk) had the highest dry matter content (12.23%) and sample 5 (100% soy beverage) had the lowest (8.20%). Sample 2 (75:25) had the highest total protein content (3.43%), and sample 3 (50:50) had the smallest (3.00%), while samples 1 and 5 had the same protein content (3.12%). The amount of lactic acid in the samples ranged from 0.47% in sample 5 (100% soy beverage) to 0.67% in sample 1 (100% cow's milk) and sample 2 (75:25). At the end of the fermentation, the pH was very similar in all samples and the pH value was constant, according to Farnworth *et al*. (*15*). Yoghurt viscosity is influenced by factors such as milk composition, heat treatment, standardization method, selection of microbial culture, inoculum quantity, temperature and duration of fermentation (*23*). The viscosity of all yoghurt samples on the first day at the initial shear rate of 100 s^-1^ ranged from 0.25 to 0.36 Pa·s, and then it began to drop abruptly at other shear rates. Sample 1 had the highest viscosity, while samples 3 and 5 show almost equal viscosity at all shear rates. By analyzing the mean values of the physical and chemical characteristics of different samples at the end of the fermentation, statistically significant differences were determined using ANOVA or Duncan's test.

**Table 1:** Physicochemical characteristics of probiotic beverage samples after fermentation

Parameter	Sample				
1	2	3	4	5
pH	(4.57±0.08)^ab^	(4.49±0.05)^bc^	(4.47±0.02)^c^	(4.57±0.04)^ab^	(4.59±0.02)^a^	
Titratable acidity/%	(0.675±0.002)^a^	(0.67±0.01)^a^	(0.584±0.003)^b^	(0.53±0.01)^c^	(0.472±0.001)^d^	
*η*/(Pa·s)	(0.36±0.04)^a^	(0.27±0.07)^c^	(0.2±0. 2)^d^	(0.31±0.04)^b^	(0.25±0.02)^d^	
*w*(protein)/%	(3.121±0.002)^c^	(3.43±0.02)^a^	(3.00±0.06)^d^	(3.169±0.001)^b^	(3.119±0.003)^c^	
*w*(fat)/%	(2.96±0.05)^a^	(2.5±0.1)^b^	(2.0±0.1)^c^	(1.5±0.1)^d^	(1.3±0.2)^d^	
*w*(sugar)/%	(5.351±0.002)^a^	(4.36±0.01)^b^	(4.27±0.06)^c^	(3.331±0.001)^d^	(3.161±0.003)^e^	
*w*(ash)/%	(0.76±0.01)^a^	(0.72±0.01)^b^	(0.69±0.02)^c^	(0.62±0.01)^d^	(0.623±0.001)^d^	
*w*(moisture)/%	(12.23±0.06)^a^	(11.0±0.2)^b^	(9.92±0.03)^c^	(8.6±0.1)^d^	(8.20±0.01)^e^	
*γ*(lactic acid)/(mg/100 mL)	(1003.0±0.6)^a^	(713.8±57.2)^b^	(756.3±35.4)^c^	(660.1±13.0)^e^	(704.4±42.6)^cd^	
***γ*(acetic acid)/** (mg/100 mL)	(9.0±1.3)^d^	(11.0±2.0)^d^	(18.4±0.4)^b^	(17.0±1.0)^c^	(27.7±3.3)^a^	
***γ*(citric acid)/** (mg/100 mL)	(72.8±13.6)^d^	(86.8±7.3)^cd^	(104.2±7.3)^c^	(123.0±2.2)^b^	(138.8±9.8)^a^	

Samples (in %): 1=cow's milk 100, 2=cow's milk 75+soy beverage 25, 3= cow's milk 50+soy beverage 50, 4=cow's milk 25+soy beverage 75, 5=soy beverage 100. Data represent mean value±S.D., *N*=3. Values with different letters in superscript in the same row are significantly different according to Duncan's multiple range test

Lactic, citric and acetic acids were identified at the end of fermentation in dairy products from cow's milk and soy beverage. Table 1 shows the production of organic acids in drinks with different ratios of cow's milk and soy beverage with probiotic strain La5. Lactic acid is important in the production of high-quality fermented milk and appropriate concentrations are required to provide the required taste with minimal sinterisation during storage (*24*). As it can be seen from Table 1, lactic acid was the dominant acid in sample 1 (100% cow's milk), while in the samples containing soy beverage the concentrations of lactic acid were similar, *i.e*. it decreased with the decrease of the amount of cow's milk. Previous studies have shown that some milk cultures cannot produce adequate levels of lactic acid in soy beverage (*25*, *26*). However, just by adding cow's milk to a soy beverage, the ability of microorganisms in the fermented yoghurt is improved so they can produce lactic acid (*25*). The concentration of lactic acid at the end of the fermentation in the analyzed samples was 660.1-1003.0 mg/100 mL, which is consistent with the research of other authors (*27*), where values at the end of fermentation ranged from 589 to 965 mg/100 g (*28*). Also, La Torre *et al*. (*29*) found approx. 1140 mg of lactic acid in 100 g of freshly prepared yoghurt, which remained constant over 20 days. However, Cruz *et al*. (*30*) found 128 mg of lactic acid per 100 mL on the first day, which increased to 306 mg per 100 mL during 28 days. Dominant acid in soy beverage sample in the present work was acetic acid, which is consistent with the research of other authors (*29*, *31*). High concentrations of acetic acid in yoghurts are generally connected with fermentation of lactose to lactic acid, and this is called the heterofermentative pathway of lactose produced by strains of bifidobacteria. La Torre *et al*. (*29*) recorded a significant increase of acetic acid concentration in fermented milk samples and yoghurts due to the addition of probiotic bacteria (*L. acidophilus, B. bifidum, B. lactis, B. longum* and/or *B. infantis*), with higher values in fermented milk than in yogurt samples. Variance analysis showed statistically significant differences in the organic acid content among different samples at the end of fermentation.

### Sugar content in probiotic beverages

Table 2 shows the content of sugars (glucose, sucrose, raffinose, stachyose, galactose and lactose) in probiotic beverages with different ratios of cow's milk and soy beverage at the end of fermentation. In fermentation with probiotic strain La5, the activity of microorganisms in the culture as a result of lactose transformation resulted in a lower pH value. The lactose content was lower in samples 3 (1.98 g/100 g) and 4 (1.05 g/100 g), with higher volume ratio of soy beverage, than in the sample containing pure cow's milk 1 (3.06 g/100 g). Soy oligosaccharides are defined as non-digestible sugars apart from sucrose. Many studies have attempted to reduce the oligosaccharide content in soy or soy products by using processing techniques such as soaking, cooking, germination, fermentation and enzyme treatment. For example, Wang *et al*. (*32*) reduced more than 80% stachyose and 50% raffinose in soy beverage by fermentation. They found the following oligosaccharides in their soy beverage (in %): raffinose 0.70, stachyose 3.79 and sucrose 3.61. Table 2 shows that these oligosaccharides (rafinose, stachyose and sucrose), the main soybean sugars during fermentation, were well transformed in pure soy beverage sample, as well as in combinations of cow's milk and soy beverage used in this work.

**Table 2:** Sugar mass fraction in samples at the end of fermentation with *Lactobacillus acidophilus* and yoghurt culture (YF-L811)

Sugar	Sample				
1	2	3	4	5
*w*(sugar)/(g/100g)				
Glucose	(1.80±0.40)^a^	(1.35±0.06)^b^	(0.53±0.01)^c^	(0.25±0.02)^d^	(0.04±0.02)^e^
Sucrose	n.d.	(0.24±0.07)^cd^	(0.27±0.05)^c^	(0.55±0.01)^ab^	(0.65±0.20)^a^
Raffinose	n.d.	(0.07±0.006)^d^	(0.08±0.001)^c^	(0.09±0.01)^b^	(0.11±0.02)^a^
Stachyose	n.d.	(0.04±0.02)^b^	(0.043±0.003)^b^	(0.04±0.08)^b^	(0.041±0.005)^b^
Galactose	(1.73±0.30)^a^	(1.30±0.05)^b^	(0.63±0.020)^c^	(0.27±0.05)^d^	(0.01±0.01)^e^
Lactose	(3.06±0.20)^a^	(2.26±0.10)^b^	(1.98±0.10)^c^	(1.05±0.06)^d^	n.d.

Samples (in %): 1=cow's milk 100, 2=cow's milk 75+soy beverage 25, 3=cow's milk 50+soy beverage 50, 4=cow's milk 25+soy beverage 75, 5=soy beverage 100. Data represent mean value±S.D., *N*=3. N.d.=not detected. Values with different letters in superscript in the same row are significantly different according to Duncan's multiple range test

Bordignon *et al*. (*33*) found that lactic acid bacteria metabolize raffinose in soy beverage, unlike yoghurt cultures, which did not affect the decrease of raffinose and stachyose during the growth in soy beverage. They proved that raffinose was essentially metabolised by strains of lactic acid bacteria, which the results of this research also confirm. The variance analysis showed statistically significant differences among the content of sugar in all samples at the end of the fermentation.

### Fatty acid profiles of probiotic beverages determined with GC-MS method

The fatty acid profiles of the drinks from fermented cow's milk and soy beverage were qualitatively and quantitatively different (Table 3). The content of saturated fatty acids ranged from 22.20 to 82.70%, while those of unsaturated fatty acids ranged from 22.31 to 77.80%. Depending on the volume ratio of cow’s milk and soy beverage, the ratio of polyunsaturated fatty acids varied from 15.50 to 65.90%. The lowest content of saturated fatty acids was recorded in the sample of pure soy beverage, and in the samples that had higher volume ratio of soy beverage. By analyzing the fatty acid content, linolenic acid (C18:2n-6) was found to be the predominant unsaturated fatty acid in soy beverage samples and in the combination of cow's milk and soy beverage where soy beverage volume ratio was higher. The mass fraction of linolenic acid (C18:2n-6c) was on average: 15.50 (sample 2), 27.20 (sample 3), 38.20 (sample 4) and 65.90% (sample 5), while of oleic acid it was from 11.90 to 22.31%. These results are similar to those in the research of soy and soy products by Peñalvo *et al*. (*34*) and Ivanov *et al*. (*35*).

**Table 3:** Fatty acid mass fractions and health lipid indices in fermented beverage samples

*w*(fatty acid)/%	Sample of fermented beverage					
1	2	3	4	5
C8	3.06^a^	2.30^b^	n.d.	1.10^c^	n.d.
C10	6.53^a^	4.90^b^	3.50^c^	2.50^d^	n.d.
					
C12	7.20	5.40^a^	4.00^b^	2.70^c^	n.d.
C13	n.d.	n.d.	n.d	n.d.	n.d.
C14	20.80^a^	15.60^a^	11.60^b^	7.90^c^	n.d.
C14:1	n.d.	n.d.	n.d.	n.d.	n.d.
C15	n.d.	n.d.	n.d.	n.d.	n.d.
C16	33.40^a^	26.80^b^	22.70^c^	17.70^d^	7.00^e^
C18	11.70	9.70^a^	9.80^a^	9.50^b^	3.50^c^
C18:1n9c	22.31^a^	19.70^b^	16.90^c^	13.30^d^	11.90^e^
C18:1n9t	n.d.	n.d.	n.d.	n.d.	n.d.
C18:2n6c	n.d.	15.50^d^	27.20^c^	38.20^b^	65.90^a^
C18:2n6t	n.d.	n.d.^a^	n.d.	n.d.	n.d.
C18:3n6	n.d.	n.d.^a^	n.d.	n.d.	n.d.
C21	n.d.	n.d.^d^	4.30^c^	6.10^b^	11.70^a^
The sum of fatty acids					
SFA	82.70^a^	64.70^b^	55.90^c^	47.50^d^	22.20^e^
MUFA	22.31^a^	19.70^b^	16.90^c^	13.30^d^	11.90^e^
PUFA	n.d.	15.50^d^	27.20^c^	38.20^b^	65.90^a^
Ratios and indices					
MUFA/SFA	0.27^c^	0.30^b^	0.30^b^	0.27^bc^	0.53^a^
PUFA/SFA	0.00	0.23^d^	0.48^c^	0.78^b^	2.96^a^
USFA/SFA	0.27^e^	0.54^d^	0.78^c^	1.06^b^	3.50^a^
DFA	34.01^e^	44.90^d^	53.90^c^	61.00^b^	81.30^a^
AI	5.75^a^	2.81^b^	1.79^c^	1.14^d^	0.14^e^
TI	5.91^a^	1.12^b^	0.58^c^	0.34^d^	0.06^e^

Samples (in %): 1=cow's milk 100, 2=cow's milk 75+soy beverage 25, 3=cow's milk 50+soy beverage 50, 4=cow's milk 25+soy beverage 75, 5=soy beverage 100. N.d.=not detected; SFA=saturated fatty acid, MUFA=monounsaturated fatty acid, PUFA=polyunsaturated fatty acid, AI=index of atherogenicity, TI=thrombogenicity index, DFA=desirable fatty acid ratio. Values with different letters in superscript in the same row are significantly different. ANOVA (p<0.05) with Duncan's *post-hoc* test

Data from Table 3 show that unsaturated fatty acids dominate in the soy beverage sample, while in other samples the amount of saturated fatty acids is increased by increasing the volume ratio of cow's milk to soy beverage. Palmitic acid is most common in the sample of pure cow's milk and the samples with higher volume ratio of cow's milk. Palmitic acid is one of the causes of increased cholesterol levels in the blood, while oleic acid has a positive effect on the human body (*36*). As a result of the different composition of fatty acids, milk lipids are characterized by a significantly different health lipid index including atherogenic index (AI), thrombogenic index (TI), desirable fatty acid ratio (DFA) and USFA/SFA ratio (Table 3). Products containing only soy beverage had the best health lipid index (AI=0.14, TI=0.06 and 81.30% DFA). The mean PUFA/SFA ratio recommended by the UK's Health Ministry is more than 0.45, and WHO/FAO experts have issued guidelines for ’balanced nutrition’ with the proposed PUFA/SFA ratio above 0.4 (*37*-*39*). From this aspect, all samples except sample 1 and 2 had favourable PUFA/SFA ratios (Table 3). In this study, samples of fermented beverages had a higher content of monounsaturated and polyunsaturated fatty acids. Analysis of variance of the samples of fermented beverages revealed a statistically significant difference in the fatty acid content. The increase in the content of monounsaturated and polyunsaturated fatty acids can be attributed to the soy beverage used in the production of probiotic beverages. This applies in particular to soy beverages and to those with a higher volume ratio of soy beverage in the starting mixture. Also, according to other research (*40*), probiotic bacteria can improve the fatty acid profile in fermented beverages, ensuring dairy products of added value. Because of this, it is possible to produce fermented beverages from soy beverage or in combination with cow's milk with potentially positive effects on human health.

### Results of SDS-PAGE analysis of fermented beverages

**Fig. 2:**
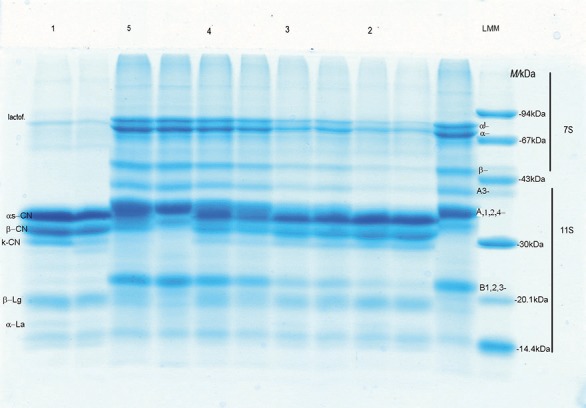
SDS electropherograms of soluble dairy protein beverages at the end of fermentation. Lane 1=100% cow's milk, lane 2=75% cow's milk+25% soy beverage, lane 3=50% cow's milk+50% soy beverage, lane 4=25% cow's milk+75% soy beverage, and lane 5=100% soy beverage, α`-, α- and β-subunits of β-conglycinin (7S). A=acid and B=base glycinin (11S), LMM=low-molecular-mass protein marker, laktof.=lactoferrin, La=lactalbumin, Lg=lactoglobulin, CN=casein

The polypeptide composition of soluble milk proteins from cow's milk and soy beverage was analyzed by SDS-PAGE (Fig. 2). The relative polypeptide compositions of soluble proteins are shown in Table 4.

**Table 4:** Polypeptide composition of milk beverage protein as a function of change in the different volume ratios of cow's milk and soy beverage

Sample	β-conglycinin (7S)				Glycinin (11S)								

α^'^	α	β	α+ α^'^	A3	αs-CN and A7,6	A1,2,4	B1,2,3,4	acidic	basic	7S	11S	11S/7S
*w*(polypeptide)/%												
2	2.85^d^	6.26^cd^	2.52^bc^	9.11^d^	3.18^d^	26.14^a^	2.53^c^	4.62^d^	5.71^d^	4.62^d^	11.63^d^	10.33^d^	0.88^cd^
3	4.75^c^	6.85^c^	2.54^bc^	11.60^c^	3.84^c^	25.03^b^	4.09^bc^	6.29^c^	7.93^c^	6.29^c^	14.14^c^	14.22^c^	1.00^c^
4	6.35^b^	8.34^b^	2.88^b^	14.69^b^	5.82^b^	24.01^c^	4.82^b^	9.67^b^	10.63^b^	9.67^b^	17.57^b^	20.30^b^	1.15^b^
5	8.23^a^	9.45^a^	7.86^a^	17.69^a^	7.01^a^	9.25^d^	18.33^a^	12.10^a^	25.33^a^	12.11^a^	25.55^a^	37.44^a^	1.46^a^

Samples (in %): 1=cow's milk 100, 2=cow's milk 75+soy beverage 25, 3=cow's milk 50+soy beverage 50, 4=cow's milk 25+soy beverage 75, 5=soy beverage 100. Mean values in the same column with diﬀerent letters in superscript are signiﬁcantly diﬀerent (p<0.05)

The SDS-PAGE electrophoresis of soluble proteins in beverages (Fig. 2) prepared from cow's milk with strain La5 (sample 1) reflects a typical polypeptide composition, which is a characteristic of products prepared from cow's milk thermally treated at high temperatures. The obtained results entirely correspond to the results of Jovanovic *et al*. (*41*) and Barac *et al*. (*42*). There are five polypeptide fractions that dominate the obtained electrophoregram: αs-CN, β-CN and κ-CN, as well as fractions of dominant serum proteins β-lactoglobulin and α-lactalbumin, which make 75.61% of the detected polypeptides. In this case, dominant fractions were αs-CN (27.66%) and β-CN (20.49%), as shown in Table 4. In addition, the presence of pale strips of high molecular mass fractions (>80 000) in the electrophoregram of sample 1 can be observed (Fig. 2), representing 4.96% of all detected polypeptides. Since extreme denaturing conditions were used in the electrophoretic analysis, the presence of these fractions indicates stable complex of milk proteins that could not be degraded under such conditions. UHT-sterilized cow’s milk was used and it is well known that extreme thermal treatments lead to the formation of a complex between casein and serum protein known as milk protein coaggregate (*42*). Generally speaking, the change in the relationship between cow's milk and soy beverage significantly affected the change of SDS-PAGE of the milk beverage polypeptide, both in terms of qualitative appearance and in the quantitative composition of the polypeptide. In addition, by comparing the electropherogram of all samples prepared from the different volume ratios of cow‘s milk and soy beverage, different resistance of cow's milk and soy beverage proteins depending on the applied starter culture could be observed. More precisely, it is certain that soy proteins were more sensitive to the proteolytic activity of the starter culture than milk proteins. Furthermore, various effects of starter culture on different soy protein fractions could also be observed (Fig. 2). Generally speaking, glycinin (11S) was found to be significantly more sensitive to the activity of the used bacteria than β-conglycinin (7S), which is somewhat unexpected, given that glycinin has a solid compact structure due to disulfide bonds and electrostatic and hydrophobic interactions (*43*, *44*). Greater sensitivity of this protein might be attributed to its partial thermal denaturation during soy beverage sterilization. The SDS-PAGE of the beverage prepared only from soy beverage (sample 5) was qualitatively and quantitatively typical for soy protein products and was in accordance with the results of Barać *et al.* (*45*, *46*) and Stanojevic *et al*. (*47*). This method was used to detect the dominant α`-, α- and β-subunits of β-conglycinine (7S) as well as acid A1,2,4-, A3-, A7,6-, A5- and base B1,2,3,4-glycinin (11S) subunits with molecular mass that are in accordance with the literature (*43*-*45*). By comparing the SDS electropherogram of cow's milk and soy beverage protein samples, it is clear that in the implemented electrophoretic system α-lactalbumin and the acidic A5-subunit had identical electrophoretic mobility, which might be a problem in their identification in a mixture of milk and soy polypeptides. Similar findings could be observed for milk lactoferin and α-subunit of β-conglicinin and for acid A1,2,4-subunits of glycinin and αs-CN. Moreover, the presence of the lipoxygenase peptide was not detected on the electropherogram of sample 5, indicating a satisfactory sterilization of soy milk.

Mass fractions of β-conglycinin (7S) and glycinin (11S) in beverages ranged from 11.63 to 25.55% and from 10.33 to 37.44% of the total number of extracted proteins (Table 4). Thanks to the greater sensitivity of soy protein to the proteolytic activity of the starter culture, electropherograms of all samples of cow's milk and soy beverages had dominant cow's milk proteins, especially αs-CN, which comprises 9.25-26.14% of all detected fractions with the acid A7,6-subunits. By reducing the volume ratio of soy beverage, but also by the more intensive proteolysis of soy protein, the ratio of these fractions increased. Furthermore, the most susceptible were the dominant acidic A1,2,4- subunits of glycinin, ranging from 2.53 (sample 1) to 18.33% (sample 5) (Table 4). For example, in a sample prepared from a mixture of cow's milk and soy beverage in the volume ratio 25:75, there was only 4.82% fractions with A1,2,4-subunits (sample 4). In contrast to these subunits, the base subunits of glycinin were found to be much more stable and ranged from 4.62 to 12.11%. The higher stability of the base subunits could be attributed to their position in the molecule. These subunits, being more hydrophobic than acids, are located inside the molecules and thus are less available to enzymes (*48*). The protein fraction analysis showed higher content of 11S than of 7S proteins in soybean products as well as in combinations of cow’s milk and soy beverage (Table 4). The protein profile obtained by the SDS-PAGE showed that there was a statistically significant difference in the mass fractions of individual protein fractions at the end of fermentation of cow's milk and soy beverage using a probiotic starter culture.

### Sensory analysis and product acceptability

Table 5 gives the results of the sensory analysis of the produced probiotic beverages. After the first day of cold storage at 4 °C, the sample obtained from 100% cow's milk (sample 1) achieved the highest sensory score, and was followed by samples 2 (75% cow's milk+25% soy beverage) and 3 (50% cow's milk+50% soy beverage), while sample 5 (100% soy beverage) received the lowest scores. The results of sensory analysis of probiotic beverages decreased with the increasing volume ratio of soy beverage. Such results clearly point to poorer sensory properties of soy beverage, because the total score of fermented beverage was almost proportional to the soy beverage ratio. However, although fermentation improves the taste of soy beverage, the undesired beany flavour and the yellowish colour of the product were still present, causing lower scores in sensory analysis. These results are in accordance with the research by Silva *et al*. (*16*). Mixing cow's milk with soy beverage significantly improved the sensory properties of the product. In sample 2 (75% cow milk+25% soy beverage), the sensory properties of odour and taste improved compared to the sample with only soy beverage.

**Table 5:** Sensory evaluation and consumer acceptability of probiotic beverages after the first day of storage at 4 °C

Property	Sample of fermented beverage				
1	2	3	4	5
Flavour (max 12)	(10.5±0.4)^a^	(8.6±2.4)^b^	(8.1±2.1)^b^	(7.4±2.8)^bc^	(6.2±2.8)^c^
Odour (max 2)	(1.9±0.2)^a^	(1.5±0.5)^b^	(1.4±0.6)^b^	(1.4±0.6)^b^	(1.4±0.7) ^b^
Appearance (max 1)	(0.99±0.03)^a^	(0.9±0.1)^a^	(0.9±0.1)^a^	(0.9±0.2)^a^	(0.8±0.2) ^a^
Colour (max 1)	(1.0±0.0)^a^	(0.9±0.2)^a^	(0.9±0.1)^a^	(0.8±0.2)^a^	(0.8±0.2)^a^
Consistency (max 4)	(3.7±0.4)^a^	(3.4±0.7)^ab^	3(.4±0.6)^ab^	(3.3±0.7)^ab^	(3.3±0.7)^b^
Total (max 20)	(18.2±1.7)^a^	(15.3±3.6)^b^	(14.6±3.3)^b^	(13.6±3.9)^b^	(12.7±4.0)^b^
Acceptability score	1	2	3	4	5
x	7.9^a^	7.1^ab^	6.7^b^	5.2^c^	3.5^d^
S.D.	0.7	1.5	1.5	2.1	2.2
Desirability/%	100.00^a^	93.00^ab^	75.00^c^	33.33^d^	13.33^e^
CV	9.28	20.67	22.67	40.69	61.18

Samples (in %): 1=cow's milk 100, 2=cow's milk 75+soy beverage 25, 3=cow's milk 50+soy beverage 50, 4=cow's milk 25+soy beverage 75, 5=soy beverage 100. Data represent mean value±S.D., *N*=3. Different letters in superscript indicate statistically significant differences among mean values±S.D. according to Duncan's multiple range test. x=mean, S.D.=standard deviation, CV=variability coefficient

**Fig. 3:**
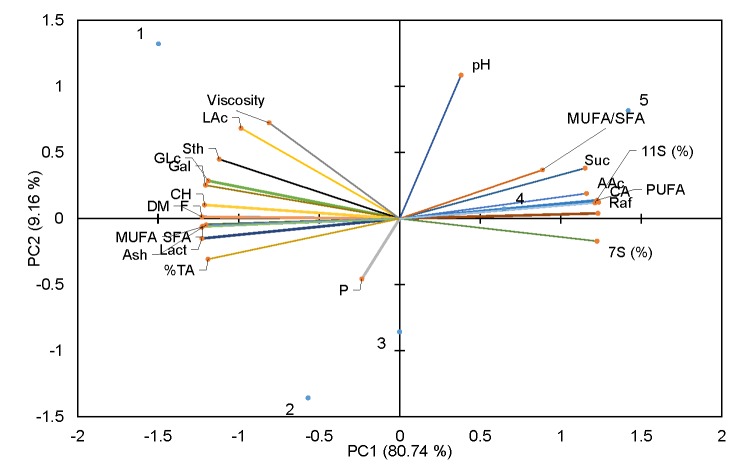
Principal component analysis of the chemical composition of cow's milk and soy milk probiotic beverages. Samples (in %): 1=cow's milk 100, 2=cow's milk 75+soy beverage 25, 3=cow's milk 50+soy beverage 50, 4=cow's milk 25+soy beverage 75, 5=soy beverage 100. SFA=saturated fatty acid, MUFA=monounsaturated fatty acid, PUFA=polyunsaturated fatty acid, TA=titration acidity, CH=carbohydrates, DM=dry matter; LAc=lactic acid, F=fats, P=proteins, Raf=raffinose, Suc=sucrose, CA=citric acid, Gal=galactose, Glc=glucose, Sth=stachyose, 11S=glycinin, and 7S=β-conglycinin

Based on the data determined by the hedonistic scale (Table 5), basic statistical parameters (mean value, standard deviation, variability coefficient) as well as the percentage of desirability and undesirability were calculated. Samples 1 (100% cow's milk) and 2 (75% cow's milk+25% soy beverage) were more desirable (93 to 100%) than samples 3 (25% cow's milk+75% soy beverage), 4 (50% cow's milk+50% soy beverage) and 5 (100% soy beverage), where desirability values ranged from 13.33 to 75.00%. Sample with 100% soy beverage (sample 5) and samples containing 50 and 75% soy beverage (samples 3 and 4) were not acceptable as their average score was less than 7 (x=3.5, 5.2 and 6.7). Based on the acceptance test of the analyzed samples, the combination of cow's milk with soy beverage up to 25% does not change the sensory properties of cow's milk, so for purposes of fermented beverage production that ratio of cow's milk and soy beverage was the most acceptable to consumers. On the other hand, soy beverage dominates the taste when it is present in higher volume ratios than cow's milk and such product was not acceptable to our consumers. Variance analysis (Table 5) showed that there were statistically significant differences among the analyzed samples of probiotic beverages. In order to confirm that samples were statistically different, the Duncan’s multiple-range test was performed. Thereby, it could be noticed that samples 1 and 2 were different from other samples. Analysis of the variances of physicochemical parameters showed that there were statistically significant differences among the tested samples of fermented beverages with different proportions of cow's milk and soy beverage. The analysis of the main components (PCA) was performed with the aim of studying the interconnection among the different variables, which in this case are physicochemical parameters. PCA was carried out on the results obtained from the produced fermented cow's milk and soy beverage. The first major component (PC1) included 80.74% of the total variability of the data, the second major component (PC2) was 9.16%. The results of PCA, precisely the mutual projections for the first two components, are presented in Fig. 3. According to the obtained results of fermented beverages by PC1, the physicochemical parameters that correlate the best are the amounts of PUFA, MUFA/SFA, 11S, acetic acid and sucrose (sample 5), and citric acid and 7S (sample 4). According to PC2, physicochemical parameters that are in positive correlation are the amounts of dry matter, fat, carbohydrates, ash, lactic acid, glucose and galactose, and viscosity, and the mass fractions of proteins, lactose, ash, MUFA and SFA (samples 1, 2 and 3).

## CONCLUSIONS

Functional probiotic beverages were successfully produced from different volume ratios of cow's milk and soy beverage using the probiotic strain of *Lactobacillus acidophilus* LA-5 with yoghurt culture. The number of probiotic bacteria in all samples of the produced beverages were in the range 7.52-8.20 log CFU/mL, which was above the generally accepted probiotic minimum (10^6^ CFU/mL), so the produced samples could be considered as probiotic products. The content of basic nutrients was similar, but the content of lactose decreased as the volume ratio of soy beverage increased. Among all acids, lactic acid was the most prevalent organic acid in all samples (660.1 to 1003.0 mg/100 mL). Fatty acid profiles of fermented beverages were characterised by higher contents of monounsaturated and polyunsaturated fatty acids, which can justify the production of fermented soy beverage or a combination of cow’s milk and soy beverage with potential positive effects on human health. The content of oligosaccharides, the main sugar of soya, was low, which was probably the result of fermentation process, with sugars being well transformed into lactic acid. Protein profiles obtained by the SDS-PAGE showed a statistically significant difference in the proportion of individual protein fractions at the end of fermentation of cow's milk and soy beverage using the probiotic starter culture. The type and volume ratio of the liquid used in the mixture mainly influenced the sensory properties of the samples. Mixing cow's milk with soy beverage improved the sensory properties of the product, especially the odour, flavour and colour. In general, all of the conducted analyses of the produced fermented beverages indicated that soy beverage ratios up to 50% were optimal for industrial-scale production of functional beverages of acceptable sensory characteristics containing probiotic bacteria and valuable soy proteins.
